# Mechanical Properties and Gamma Radiation Transmission Rate of Heavyweight Concrete Containing Barite Aggregates

**DOI:** 10.3390/ma15062173

**Published:** 2022-03-15

**Authors:** Baitollah Badarloo, Petr Lehner, Rooholah Bakhtiari Doost

**Affiliations:** 1 Department of Civil Engineering, Qom University of Technology (QUT), Qom 37181-46645, Iran; bakhtiaridoost@qut.ac.ir; 2 Faculty of Civil Engineering, Department of Structural Mechanics, VSB-Technical University of Ostrava, Ludvíka Podéště 1875/17, 708 33 Ostrava-Poruba, Czech Republic

**Keywords:** heavyweight concrete, gamma radiation, attenuation coefficient, barite aggregates

## Abstract

The primary objective of this research was to study the transmission of gamma radiation from heavyweight concrete containing barite aggregates. For this purpose, cylindrical and cubic specimens were produced for 10 mix designs. The mix designs containing different percentages of barite aggregates were calculated; five mix designs were also calculated for the compressive strength of 25 MPa, while five of them were designed for the compressive strength of 35 MPa to study the influence of the compressive strength rate on the reduction in gamma radiation transmission. The results indicated that both compressive and tensile strength was decreased by increasing the ratio of barite aggregates. The rate in reduction of compressive strength and especially tensile strength in concrete C35 was less than in concrete C25. The use of barite aggregates increased the attenuation coefficient of concrete. The attenuation coefficient in C35 concrete increased more than that in C25 upon increasing the amount of barite aggregate. By increasing the thickness of concrete with different percentages of barite, the rate of radiation loss in different samples was closer. The difference in the rate of radiation loss at a thickness of 150 mm was not much different from that at a thickness of 100 mm, whereas it was considerably decreased at a thickness of 300 mm. The test results indicated that the reduction in the gamma transmission rate is significantly dependent on the density of concrete.

## 1. Introduction

Today, with the progress in nuclear technology, the application of radiation is becoming widespread in some areas, including the agricultural, industrial, and medical fields. Gamma and X-ray radiation occurs as a result of atomic reactions. These kinds of radiation have high penetration capability in material, and they need to be attenuated using a proper method and material due to the harmful effects of nuclear radiation on humans. The design of radioactive radiation shields is one of the significant concerns in nuclear science.

Different materials such as lead, graphite, iron, polyethene, and concrete are used to protect against nuclear radiation. Concrete is one of the most appropriate options, and it is commonly used for such purposes. According to the American concrete institute ACI 211 [1], heavyweight concrete can be used as a protecting shield against radiation. This concrete is also used to decrease the thickness of the protecting shields. Another use of heavyweight concrete is for storing radioactive wastes [2].

Commonly, concrete with a unit weight above 2600 kg/m^3^ is categorized as heavyweight concrete. It is usually made using coarse aggregates with specific gravities above 3000 kg/m^3^ [3]. The mix design of heavyweight concrete is very similar to ordinary concrete. The mix design of heavyweight concrete is presented in ACI 304 [4].

Lead or heavy aggregates are usually used to make heavy concrete. Due to the adverse effects of lead on human health, protective shields can be created using barite, hematite, siderite, limonite, ilmenite, or serpentine containing iron ore, which is generally of high density [5,6]. The aggregates of concrete play a principal role in improving concrete shielding properties and, therefore, have good shielding properties for the attenuation of X and gamma rays [7].

Delnavaz et al. [8] experimentally studied the gamma radiation protection rate of heavy concrete samples containing barite and iron oxide aggregates. Samples with thicknesses of 100, 150, 200, and 300 mm with different participation percentages were produced, and then a radioactive gamma test was implemented. As a result of their study, it was found that samples containing 100% barite and 100% iron oxide revealed the highest linear attenuation coefficient against a cesium-137 radioactive source, about 50% and 40% higher than normal-weight concrete, respectively.

Other studies presented combinations of barite aggregate with other materials such as titanium-rich slag [9], sand, or nanomaterial [10]. Tasnim et al. [11] conducted a very detailed study of several concretes incorporating barium sulfate. These were materials with high densities of over 3300 kg/m^3^. Similarly, Daungwilailuk et al. [12] presented the results of an experimental study that included findings on the combination of barite aggregate and steel bars. The results declared that, in this combination, it was not possible to focus purely on the density of the materials as it did not guarantee the best radiation protection. Taban Shams et al. [13] made concrete shields for gamma rays using barite and hematite gradation in separate, mixed, and multilayered forms. The results showed that increasing the barite and hematite aggregates, whether separately or as a mixture, increased the characteristic compressive strength and linear attenuation coefficient (LAC), and it was found that the order of the layers in multilayered shields had no significant effect on the attenuation of gamma radiations.

Mukhtar Oluwaseun Azeez et al. [14] carried out an experimental investigation to investigate the radiation shielding performance of heavyweight concrete mixes prepared using different high-density coarse aggregates that included iron ore, steel shot, and steel slag, and radiation shielding performance was influenced by the unit weight of the concrete mixtures regardless of the type of aggregates. They found out that the mix design with 100% participation of steel shot aggregates showed the best radiation shielding performance among all the mixtures with the least required thickness.

An investigation was conducted by Mustafa Çullu et al. [15] at Gumushane University in Turkey. In their study, concrete specimens were prepared by altering different amounts of heavy aggregates. The samples were made in different concrete strength grades, and, after curing duration, radiation absorption experiments were carried out on specimens. They concluded that concrete strength grade affects the radiation attenuation coefficient in concretes created entirely by heavy aggregates, while the radiation absorption coefficients decreased upon increasing gamma energies. Süleyman Gökçe et al. [16] concluded from their research on reactive powder concrete using barite aggregate that barite increased the gamma ray mass attenuation coefficients of reactive powder concrete mixtures below 0.3 MeV and above 8 MeV energies.

An investigation on heavy concrete with different iron aggregate inclusions revealed that wave velocity measured from heavy concrete decreased with the increase in iron ore [17]. Ameri et al. [18] investigated the effect of copper slag on impact resistance, mechanical and microstructural characteristics, and gamma ray shielding of steel fiber-reinforced high-strength concrete. Khalaf et al. [19] indicated that the use of steel furnace slag in heavy concrete improves durability, mechanical properties, and radioactive ray protection. Esfahani et al. [20] showed that the use of copper slag and ground granulated blast furnace slag in heavy concrete enhanced the workability of fresh concrete, mechanical properties, and gamma ray shielding. Dąbrowski et al. [21] studied the microstructural features and mechanical properties of heavy concrete with different heavy aggregates during hardening mixes.

All this knowledge was considered within the current situation in Iran, which is on a research path toward clean nuclear energy and where barite aggregate is available. Therefore, research requirements were put forward on the amount of replacement for sand and gravel and other influences that can provide the broader knowledge needed for future infrastructure building. A final issue is the relationship of density and linear attenuation coefficient for different grades of concrete containing barite aggregate as a replacement for sand and gravel. To this final question must be added other objectives, which are the subject of the present manuscript. In the present study, the effects of specimen thickness, participation percentage of barite aggregates, and concrete strength grade on gamma radiation absorption rate were investigated. To test the gamma transmission rate and determine the mechanical properties of heavy concrete, five different mix designs were obtained by substituting 0%, 25%, 50%, 75%, and 100% normal aggregates with barite aggregates. Two concrete strength grades with the mentioned substitution rates at thicknesses of 100 mm, 150 mm, and 300 mm were produced and tested.

## 2. Materials

### 2.1. Aggregates

**Figure 1:**
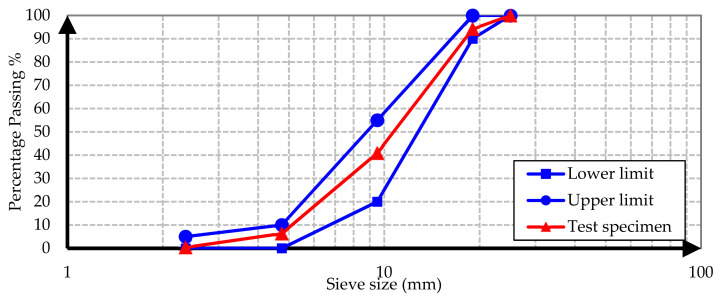
Conventional gravel sieve analysis grading curve.

**Figure 2:**
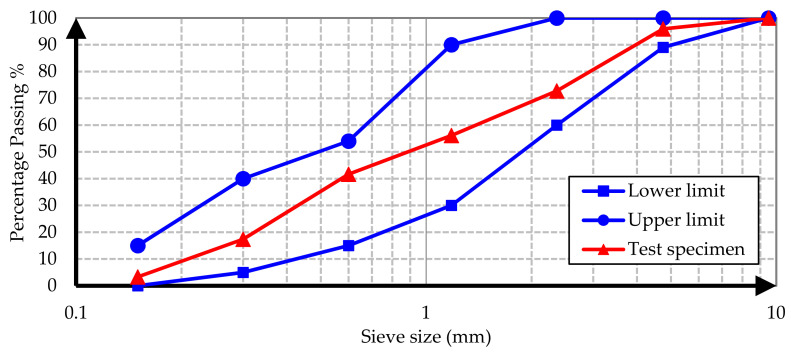
Conventional sand sieve analysis grading curve.

**Figure 3:**
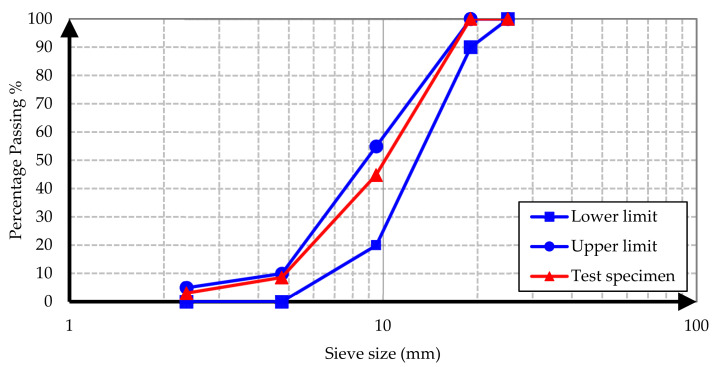
Barite gravel sieve analysis grading curve.

**Figure 4:**
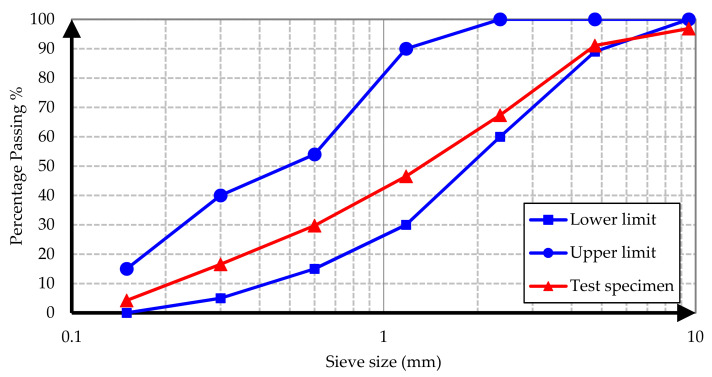
Barite sand sieve analysis grading curve.

Conventional and barite aggregates were used as aggregates in all concrete samples. The conventional aggregate is an aggregate of normal volume and weight used in the production of conventional concrete mixes. To determine the grading of aggregates, a sieve analysis test was performed on specimens from conventional and barite aggregates according to ASTM C637 [22]. The test results are illustrated in Figure 1, Figure 2, Figure 3 and Figure 4.

It is evident from the results that both conventional and barite aggregates had continuous grading. The final diagram was within the standard range, which means that the aggregates were well graded. Moreover, the nominal maximum size of coarse aggregates was 20 mm. The water absorption and specific gravity of fine and coarse aggregates are presented in Table 1.

### 2.2. Cement

Type I Portland cement with a specific gravity of 3150 kg/m^3^ was used in all the concrete mixes [23]. The chemical compositions of the cement are shown in Table 2.

### 2.3. Water

The physical and chemical properties of the utilized water are summarized in Table 3. Notably, the amounts of other ions (F, Ca, Mg, Na, K, etc.) were negligible; therefore, the values are not included in the table.

## 3. Mixing and Sample Preparation

A total of 10 mix designs were generated with different combinations of coarse aggregates. Five mixtures were designed for 25 MPa concrete strength, while five other mixtures were designed to achieve 35 MPa concrete strength. In each C25 mix design, 390 kg/m^3^ of cement was used, while C35 concrete mixtures contained 410 kg/m^3^ of cement in every mix. For all concrete mixtures, the water/cement ratio was kept constant at 0.5. Notably, mix design calculations were conducted according to the ACI-211-89 standard [1].

To study the influence of concrete specific weight on gamma radiation transmission, barite aggregates were used to replace 0%, 25%, 50%, 75%, and 100% of conventional sand and gravel by weight. Moreover, to investigate the influence of concrete thickness on gamma radiation transmission, cubic specimens with dimensions of 10 cm × 10 cm × 10 cm and 15 cm × 15 cm × 15 cm, as well as cylindrical samples with dimensions of 15 cm × 30 cm, were made. Two samples were made from each mix design, and the total number of samples was 60. Mix designs are demonstrated in Table 4 with details. The proposed concrete mixing schedule is related to the saturation state with the dry surface, while the amount of water and aggregates was modified according to the moisture content of the aggregates in the laboratory.

**Figure 5:**
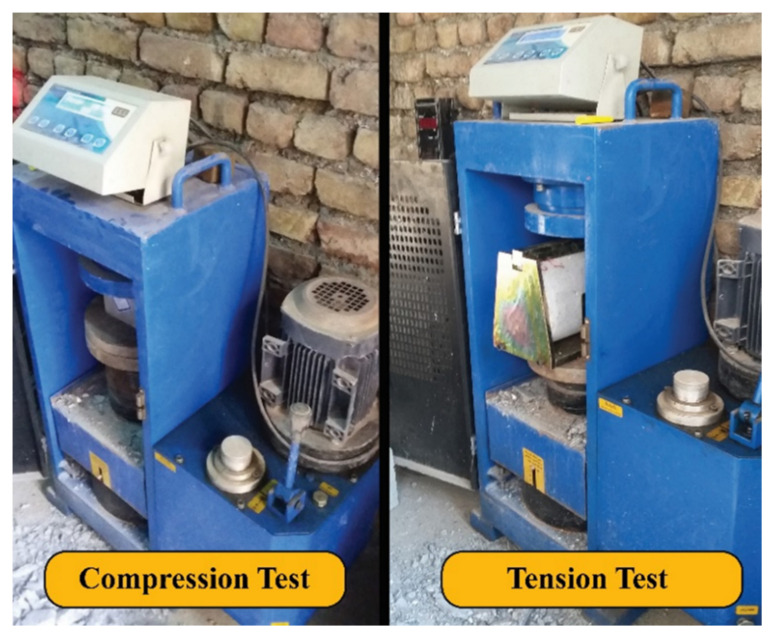
Concrete testing equipment.

The construction of concrete samples was performed in a controlled laboratory environment. Concrete constituents were weighed and then mixed in an electronic mixer. After mixing, process test specimens were cast into mentioned molds. A standard tamper was used to vibrate the samples, and each sample was compacted using the tamper method. Demolding of the specimens was done after 24 h, and then the curing process was implemented using a water tank for 28 days. The standard certified laboratory equipment shown in Figure 5 was used for testing.

The unit weight and the compressive and tensile strength of hardened concrete are summarized in Table 5. Reported values are the average values of two tests. To perform compression and tension tests, hydraulic concrete testing equipment was used with 200 tons of maximum tolerable force.

## 4. Gamma Radiation Transmission Test Setup

**Figure 6:**
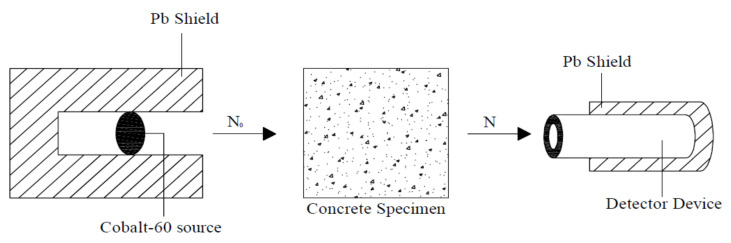
Gamma-ray transmission measurement test setup.

**Figure 7:**
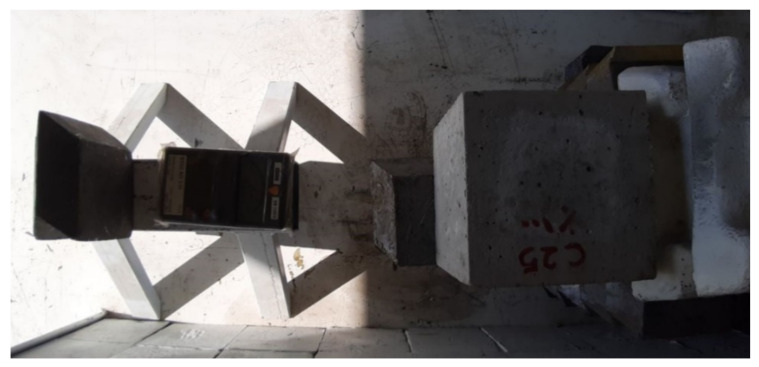
Gamma-ray transmission measurement test setup in the lab.

The number of transmitted rays through specimens with different thicknesses exposed to gamma radiation was measured by a detector device (AUTOMESS 6150AD5/H, Automess, Ladenburg, Germany). The source of gamma radiation was cobalt-60 element, and the radioactive activity of the source was 16.8 mCi. Furthermore, the intensity of radiation without shielding was measured at 520 mSv/h. The distance between the radiation source and the detector device was 55 cm. The arrangement of the test setup is presented in Figure 6 and Figure 7. 

## 5. Results, Analysis, and Discussion

### 5.1. Compressive Strength

To measure the compressive strength of concrete mix designs, cubic specimens were made as mentioned previously, and their compressive strength was tested using a standard hydraulic testing machine. By comparing the final results, it could be concluded that, by increasing the amount of barite aggregate in the mixture, the compressive strength of the produced concrete would decrease.

**Figure 8:**
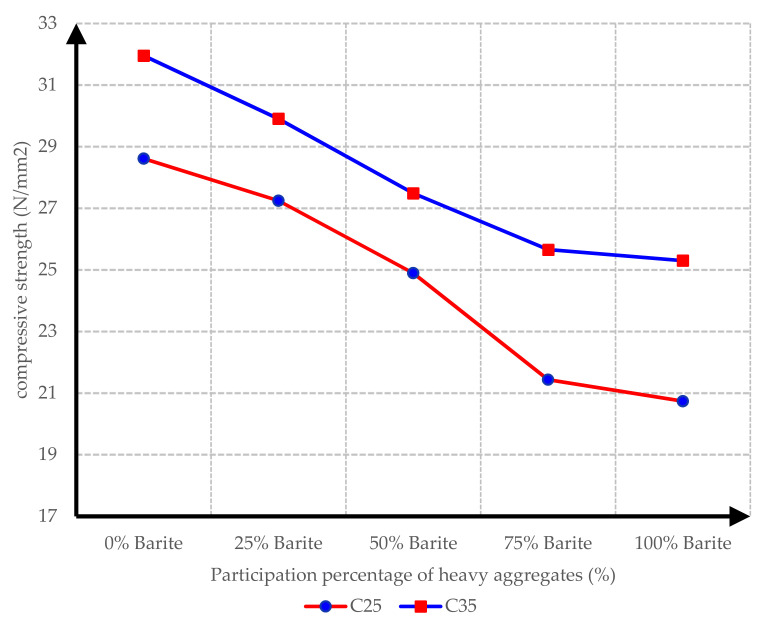
Diagram of compressive strength of concrete specimens and participation percentage of barite aggregates for C25 and C35 concrete mixtures.

The reason for this might be the weaker properties of barite aggregates in comparison with conventional aggregates. The loss of strength by increasing the participation ratio of heavy aggregates is illustrated in Figure 8. It is intelligible that, upon 100% replacement of barite aggregates, the compressive strength of C25 concrete and C35 concrete decreased by 27.52% and 20.82%, respectively. It should be noted that the required strength values of around 25 and 35 MPa were given in advance. This must be taken into account when comparing the results with other very heavy concretes with high strengths of around 45 to 50 MPa [10,12].

### 5.2. Tensile Strength

**Figure 9:**
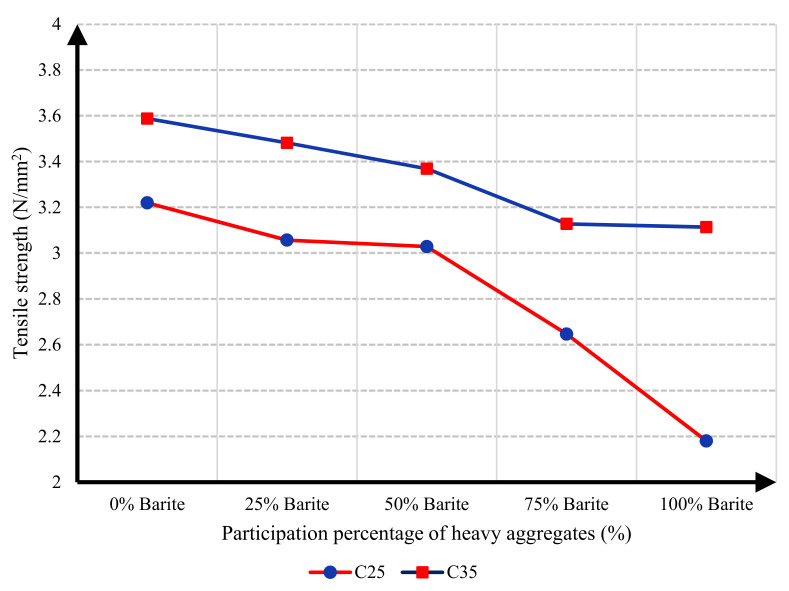
Diagram of the tensile strength of concrete specimens and participation percentage of barite aggregates for C25 and C35 concrete mixtures.

To determine the tensile strength of samples, the splitting tensile strength test (Brazilian tensile test) was performed on cylindrical specimens. As with compressive strength, the values for tensile strength also decreased upon increasing the heavy aggregates. For a better comparison, the diagram of tensile strength and the participation percentage of heavy aggregates are presented in Figure 9. Upon 100% replacement of barite aggregates, the tensile strength of C25 concrete and C35 concrete decreased by 32.30% and 13.21%, respectively.

### 5.3. Failure Mode

**Figure 10:**
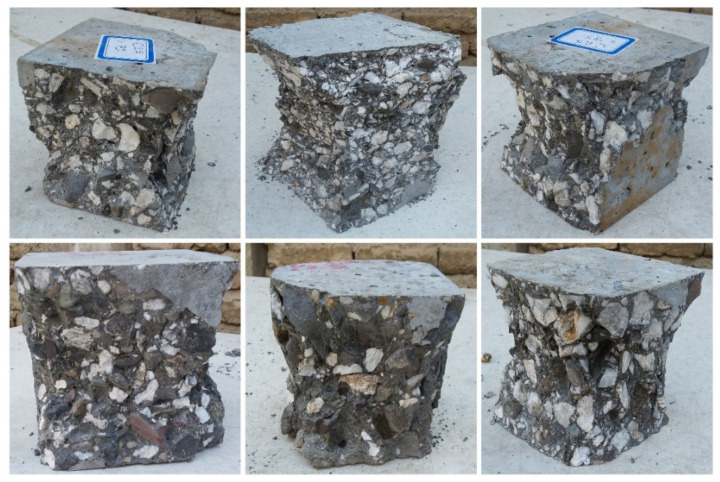
The dominant failure mode for cubic samples.

According to the BS EN 12390-3 [24] standard, most cubic samples had a semi-explosive failure mode, which is categorized as a satisfactory failure mode. As shown in Figure 10, in all cubic samples, aggregates were crushed, and failure was not propagated in the cementation matrix.

**Figure 11:**
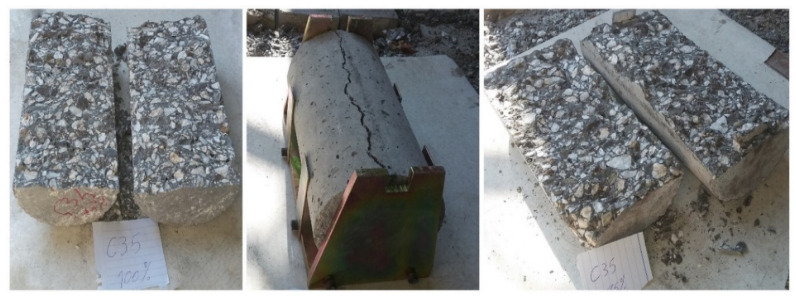
The dominant failure mode for cylindrical samples.

Moreover, after the tensile strength test, all cylindrical specimens split into two equal parts, which is categorized as an ideal failure mode for the Brazilian tensile test and a satisfactory failure mode by international standards. The dominant failure mode for cylindrical samples is shown in Figure 11.

### 5.4. Radiation Test Results

According to the Lambert law, the attenuation coefficient of the concrete samples can be calculated from Equation (1) [25,26].

(1) $$N=N_{0}e^{-\mu A},$$

where *N*_0_ denotes the photon intensities without shielding, *N* denotes photon intensities with shielding, $\mu \;\left({\mathrm{mm}}^{-1}\right)$. is the linear attenuation coefficient of the material (LAC), and *A* is the concrete thickness.

Plotting each ln(*N*_0_/*N*) versus *A* would provide a straight line, and *μ* can be determined by the slope’s value. A higher linear attenuation coefficient indicates better radiation absorptive ability.

Another significant factor in the design of radiological protection barriers is the half-value layer thickness (HVL). The half-value layer (HVL) is the minimum thickness of a material that reduces the radiation intensity to 50% of the initial radiation intensity. This thickness is inversely proportional to the attenuation coefficient (*μ*) and is calculated according to Equation (2). According to the definition of HVL, materials with lower HVL should lead to more thin walls [27].

(2) HVL=ln2/μ.

The rate of radiation loss is defined as follows:

(3) $$\mathrm{Rate}\;\mathrm{of}\;\mathrm{radiation}\;\mathrm{loss}=100\times (\frac{N_{0}-N}{N_{0}}).$$

**Figure 12:**
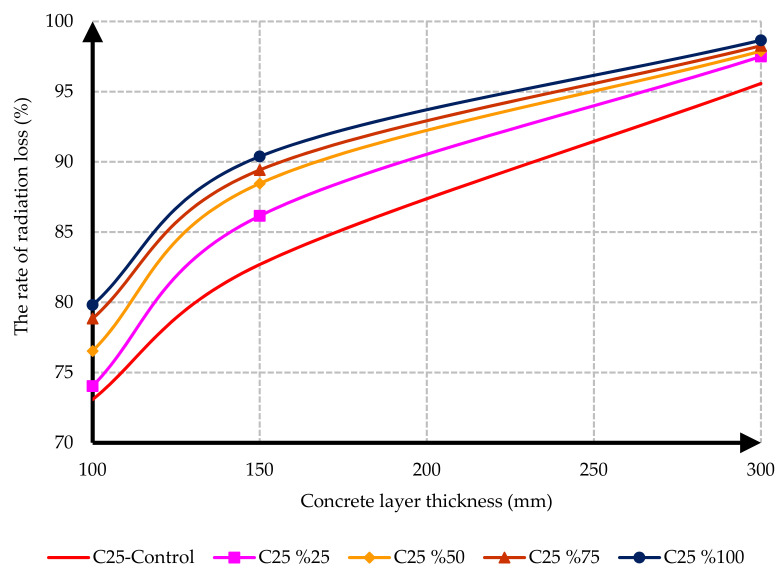
Effect of barite aggregates on the rate of radiation loss for C25 concrete mix designs.

**Figure 13:**
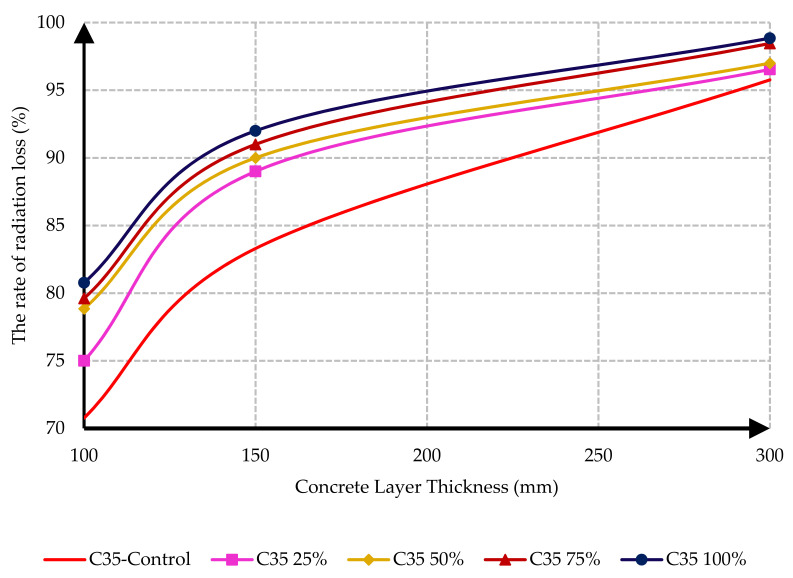
Effect of barite aggregates on the rate of radiation loss for C35 concrete mix designs.

The rate of radiation loss for the control sample and the specimens containing different percentages of barite aggregate versus layer thicknesses is presented in Figure 12 and Figure 13. As a result of the tests, the rate of radiation loss in heavy concrete samples increased with the increase in barite aggregates. By comparing C25 and C35 specimens, it is evident that, in C35 specimens, except for the control sample, the rate of radiation loss was higher than in C25 specimens. The reason for this may be the lower density of the control sample of C35 than the control sample of C25.

Another important conclusion from Figure 12 and Figure 13 is the significant role of layer thickness in decreasing the gamma radiation transmission rate. It resulted from the tests that the maximum passage rate loss occurred in the 300 mm thick layer of C35 concrete and could decrease the radiation passage rate by up to 98%.

As mentioned before, in addition to the transmission flux rate, the HVL (half-value layer thickness) is a main factor for designing and determining the thickness of the protective barrier against gamma rays.

**Figure 14:**
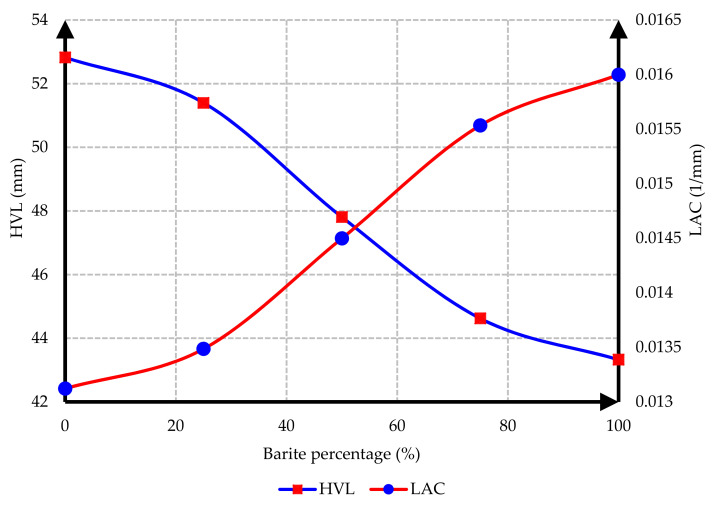
Interaction between the attenuation and the HVL for C25 concrete.

**Figure 15:**
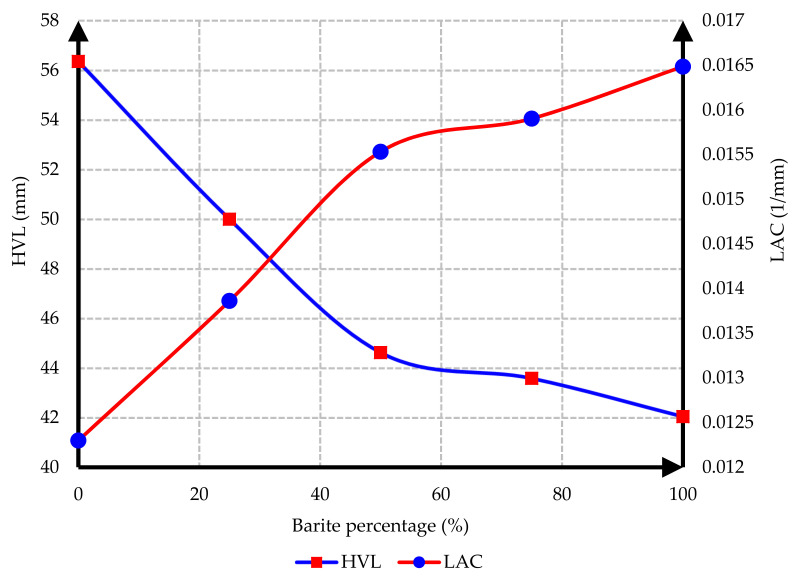
Interaction between the attenuation and the HVL for C35 concrete.

The interaction between the attenuation and the HVL for C25 and C35 concrete based on heavy aggregate participation percentages is demonstrated in Figure 14 and Figure 15.

As depicted in Figure 14 and Figure 15, the HVL decreased upon increasing the percentage of barite. Therefore, when using heavy concrete, the thickness of the protective barrier against gamma radiations would be reduced. Furthermore, the linear attenuation coefficient (LAC) increased upon increasing the percentage of barite.

**Figure 16:**
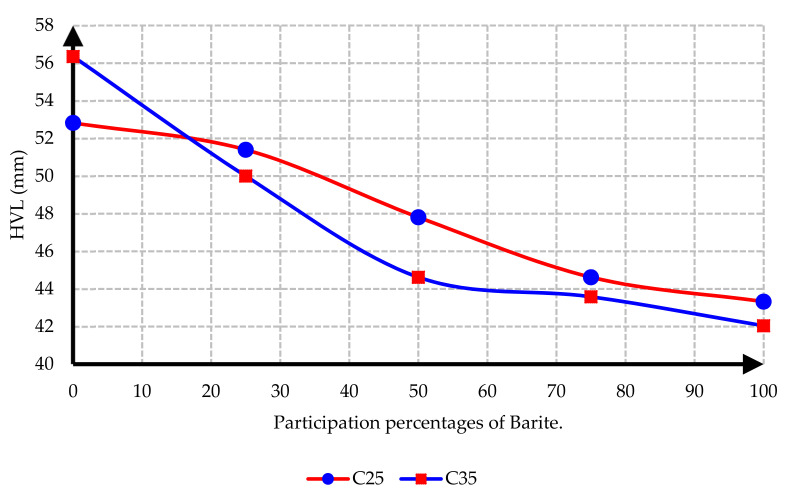
Comparison of HVL thicknesses in C25 and C35 concretes with different proportions of barite.

Figure 16 depicts the evaluation of HVL thicknesses in C25 and C35 concretes with different ratios of barite, revealing noteworthy losses in HVL values upon adding barite to concretes.

**Figure 17:**
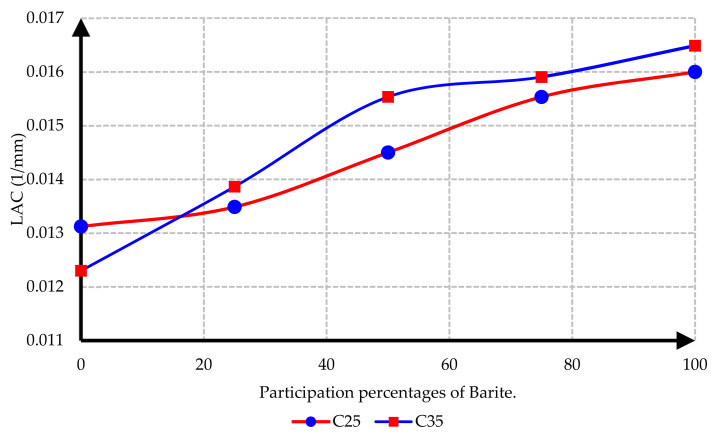
Comparison of LAC in concretes with different ratios of barite.

As depicted in Figure 17, the LAC of heavy concrete mixes increased with the increase in barite aggregates. By comparing C25 and C35 specimens, it could be concluded that the LAC in C35 concretes, except the control sample, was higher than in C25 concretes, because of the more coherent concrete matrix in C35 and its higher specific weight. The highest LAC against radiation was reported for C35 concrete containing 100% barite. A higher linear attenuation coefficient (LAC) indicates better radiation absorptive ability.

**Figure 18:**
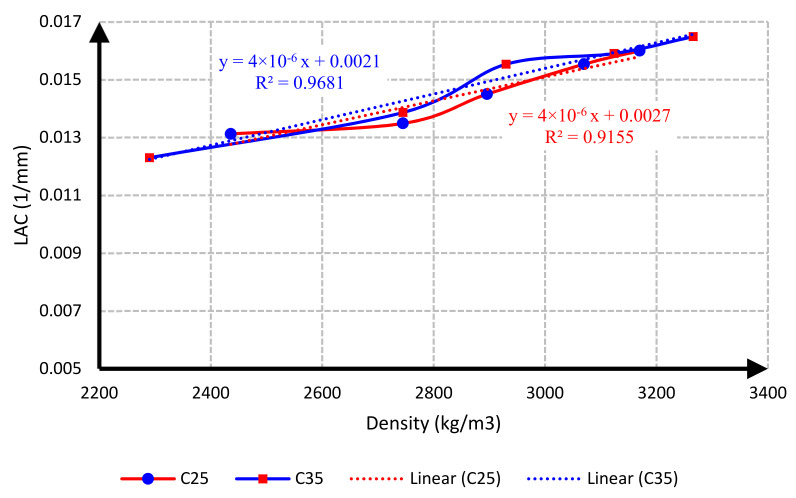
Density–LAC relationship for C25 and C35 concrete.

Figure 18 shows the LAC–density relationship of the C25 and C35 concrete samples. The best equation that corresponded to the relationship between density and LAC is the linear equation shown on the curve. A comparison of the equations obtained for concretes C25 and C35 shows that, regardless of the strength of concrete, the amount of LAC depended on the density of concrete, yielding the following relationship between density and LAC:

(4) $$\mu =4\times 10^{-6}\rho +0.0024,$$

where ρ is density in terms of kg/m^3^, and μ is linear attenuation coefficient in terms of 1/mm.

## 6. Conclusions

In this research, protection against gamma radiation was experimentally evaluated, and radiation absorption tests were performed on concrete specimens produced with ordinary aggregates and heavyweight aggregates. For this aim, concrete samples with thicknesses of 100 mm, 150 mm, and 300 mm were constructed in the lab. Participation percentages of heavy aggregates in different mix designs were selected as 0%, 25%, 50%, 75%, and 100%. The following conclusions can be drawn from this study:

1. Due to the lower hardness and strength of barite aggregates compared to ordinary aggregates, the compressive and tensile strength of concrete had an inverse relationship with the amount of barite aggregate. By increasing the amount of barite aggregate in the mixture, the rate of reduction in compressive strength and especially tensile strength in concrete C35 was less than in concrete C25.
2. Upon 100% replacement of barite aggregates, the compressive strength of C25 concrete and C35 concrete decreased by 27.52% and 20.82%, respectively, while the tensile strength of C25 concrete and C35 concrete decreased by 32.30% and 13.21%, respectively. This result shows that, according to the mixing design of C25 and C35, increasing the consumption of cement in concrete reduces the intensity of the decrease in compressive strength and especially tensile strength of concrete due to the increase in the amount of barite.
3. The rate of radiation loss in heavy concrete samples increased with the increase in barite aggregates. By comparing C25 and C35 specimens, understandably, the rate of radiation loss in C35 concrete samples was higher than in C25 concrete samples for thinner layers. For C35, as the thickness of concrete increased, the difference in the percentage of radiation transmission reduction decreased in concretes with different percentages of barite aggregates. The difference in the rate of radiation loss in concrete samples of 100 mm thickness without and with 100% barite aggregates reached about 9%. For all C25 and C35 concrete samples with a layer thickness of 300 mm, the rate of radiation loss was between 96% and 99%.
4. From the LAC calculations, it is evident that the LAC of heavy concrete mixes increased with the increase in barite aggregates, while the LAC in C35 concretes with the same percentage of barite was larger than in C25 concretes. The amount of LAC increased by 23% and 35%, respectively, in C35 and C25 concretes with 100% barite compared to samples without barite. The maximum LAC against the cobalt-60 source was recorded as 0.0165 for C35 concrete with 100% barite aggregate.
5. The HVL thicknesses in C25 and C35 concretes with different ratios of barite exhibited noteworthy losses with an increase in barite addition. The HVL decreased by 25% and 19% in C35 and C25 concretes with 100% barite compared to samples without barite.
6. For C25 and C35 concretes, regardless of the strength, the amount of LAC depended on the density of concrete. The relationship between LAC and density of heavyweight concrete made with barite was obtained as a linear equation on the basis of the results that can be used for other thicknesses.

## Figures and Tables

**Table 1:** Physical and mechanical properties of aggregates.

	Conventional Aggregates		Barite Aggregates	
	Coarse	Fine	Coarse	Fine
Water absorption (%)	1.2	2.1	0.32	0.36
Specific gravity (kg/m^3^)	1634	1760	3205	3610

**Table 2:** Physical and chemical properties of cement used.

**Chemical composition**	SiO_2_	23.50%
	Al_2_O_3_	5.80%
	Fe_2_O_3_	3.10%
	CaO	60.00%
	MgO	3.10%
	Cl	0.025%
	SO_3_	2.00%
	Loss of ignition	1.10%
	Insoluble residue	0.35%
**Physical properties**	Specific gravity	3150 kg/m^3^
	Outlet start	160 min
	End of the outlet	230 min
	Blaine	3500 cm^2^/gr
**Compressive strength of the cement**	2 days	150 kg/cm^2^
	7 days	350 kg/cm^2^
	28 days	490 kg/cm^2^

**Table 3:** Physical and chemical properties of water used.

Test Name	Unit	Results	Test Method
EC	μs/cm	132.5	Std. M. 2510.B
TDS	mg/L	92	Std. M. 2540.C
TSS	mg/L	4	Std. M. 2540.C
Cl^−^	mg/L	19.31	Std. M. 4500-Cl.B
${\mathrm{PO}}_{4}^{2-}$	mg/L	0.017	Std. M. 4500-P.E

**Table 4:** Mixture proportions of concrete (kg/m^3^).

Mix ID	W/C	Water	Cement	Normal Sand	Normal Gravel	Barite Sand	Barite Gravel
C25-CO	0.5	195	390	680	1162	0	0
C25-Barite-25%	0.5	195	390	510	872	302	547
C25-Barite-50%	0.5	195	390	340	581	604	1093
C25-Barite-75%	0.5	195	390	170	291	907	1640
C25-Barite-100%	0.5	195	390	0	0	1209	2187
C35-CO	0.5	205	410	658	1162	0	0
C35-Barite-25%	0.5	205	410	494	872	293	547
C35-Barite-50%	0.5	205	410	330	581	585	1094
C35-Barite-75%	0.5	205	410	165	291	877	1640
C35-Barite-100%	0.5	205	410	0	0	1170	2187

**Table 5:** Results of compressive and tensile strength tests and unit weight of the samples with different mixture designs.

Mix ID	Compressive Strength (N/mm^2^)	Tensile Strength (N/mm^2^)	Specific Weight (kg/m^3^)
C25-CO	28.612	3.220	2415
C25-Barite-25%	27.246	3.057	2668
C25-Barite-50%	24.898	3.029	2715
C25-Barite-75%	21.436	2.647	3080
C25-Barite-100%	20.737	2.180	3221
C35-CO	31.952	3.588	2382
C35-Barite-25%	29.905	3.482	2709
C35-Barite-50%	27.485	3.369	2916
C35-Barite-75%	25.658	3.128	3110
C35-Barite-100%	25.300	3.114	3282

## Data Availability

Not applicable.
